# Identification of Critical Amino Acids in an Immunodominant IgE Epitope of Pen c 13, a Major Allergen from *Penicillium citrinum*

**DOI:** 10.1371/journal.pone.0034627

**Published:** 2012-04-10

**Authors:** Jui-Chieh Chen, Li-Li Chiu, Kuang-Lun Lee, Wei-Ning Huang, Jiing-Guang Chuang, Hsin-Kai Liao, Lu-Ping Chow

**Affiliations:** 1 Graduate Institute of Biochemistry and Molecular Biology, College of Medicine, National Taiwan University, Taipei, Taiwan; 2 Department of Internal Medicine, National Taiwan University Hospital, Taipei, Taiwan; 3 Department of Biotechnology, Yuanpei University, Hsinchu, Taiwan; Centre de Recherche Public de la Santé (CRP-Santé), Luxembourg

## Abstract

**Background:**

Pen c 13, identified as a 33-kDa alkaline serine protease, is a major allergen secreted by *Penicillium citrinum*. Detailed knowledge about the epitopes responsible for IgE binding would help inform the diagnosis/prognosis of fungal allergy and facilitate the rational design of hypoallergenic candidate vaccines. The goal of the present study was to characterize the IgE epitopes of Pen c 13.

**Methodology/Principal Findings:**

Serum samples were collected from 10 patients with mold allergy and positive Pen c 13 skin test results. IgE-binding epitopes on rPen c 13 were mapped using an enzymatic digestion and chemical cleavage method, followed by dot-blotting and mass spectrometry. A B-cell epitope-predicting server and molecular modeling were used to predict the residues most likely involved in IgE binding. Theoretically predicted IgE-binding regions were further confirmed by site-directed mutagenesis assays. At least twelve different IgE-binding epitopes located throughout Pen c 13 were identified. Of these, peptides S16 (A^148^–E^166^) and S22 (A^243^–K^274^) were recognized by sera from 90% and 100% of the patients tested, and were further confirmed by inhibition assays. Peptide S22 was selected for further analysis of IgE-binding ability. The results of serum screening showed that the majority of IgE-binding ability resided in the C-terminus. One Pen c 13 mutant, G270A (T^261^–K^274^), exhibited clearly enhanced IgE reactivity, whereas another, K274A, exhibited dramatically reduced IgE reactivity.

**Conclusions/Significance:**

Experimental analyses confirmed *in silico-*predicted residues involved in an important antigenic region of Pen c 13. The G270A mutant of Pen c 13 has the potential to serve as an additional tool for the diagnosis/prognosis of mold allergy, and the K274A mutant, as a hypoallergenic form of the epitope, may provide a framework for the design and development of a safe and efficient therapeutic strategy for treating human allergic diseases.

## Introduction

Fungi are ubiquitous in our environment, and many have been identified as causative factors in IgE-mediated allergy. Among the fungi associated with allergic disorders, *Penicillium* sp. is one of the most frequently encountered species [1]–[4]. Previous studies identified the major allergen Pen c 13, a highly prevalent allergen secreted by *Penicillium citrinum* among patients with mold allergy [5], as a 33-kDa alkaline serine protease, and showed that it might directly contribute to sensitization and is associated with the development of asthma [6]–[9]. These effects probably result from the pathophysiological changes in the lung microenvironment, the expression of thymic stromal lymphopoietin, and the migration of antigen-presenting cells to the draining lymph node under the influence of protease [10]–[12].

Understanding of IgE-binding epitopes on allergens should help elucidate allergen-antibody interactions and may contribute to the development of more effective immunotherapeutic strategies. It has been demonstrated that immunotherapy induces strong allergen-specific IgG responses and that protective IgG antibodies are important for the success of allergen-specific immunotherapy [13]–[17]. The effectiveness of recently developed genetically engineered and synthetic allergen derivatives that have been modified to reduce their allergenic activity demonstrates the potential use of specific epitopes in immunotherapeutic approaches to allergic disease [18]–[21].

Among the strategies for identifying allergen epitopes are fragmentation of allergens, either enzymatically [3], [22] or synthetically [23], [24]; phage display [25], [26]; and various recombinant technologies [27]–[29]. Epitopes might be composed of sequential residues along the polypeptide chain (linear epitopes) or nonsequential residues from segments of the chain brought together by the folded conformation of the protein (conformational epitopes) [30], [31]. Many epitope-mapping studies of different allergens have shown that small linear epitopes bind specifically to IgE antibodies of allergic patients; moreover, the IgE-binding capacity of these allergens could be abolished by single amino acid mutations within each epitope [26], [27], [32]–[34]. Furthermore, the identification of specific residues involved in IgE antibody binding can be sped up using computer programs that predict B-cell epitopes [35]–[39].

Pharmacological agents have been the mainstay of allergy symptom management, but allergen-specific immunotherapy is the only long-lasting, allergen-specific approach for treating type I allergy and preventing its progression to severe disease manifestations [40]–[43]. An important element in the implementation of specific immunotherapy is developing a clearer understanding of the immunogenicity of allergen derivatives.

In the present study, we mapped the IgE-binding epitopes on recombinant Pen c 13 (rPen c 13) using enzymatic digestion and chemical cleavage methods, followed by dot-blotting and mass spectrometry. A concentration-response inhibition assay was performed to provide evidence of IgE binding. We also integrated results from a B-cell epitope-predicting server and molecular modeling to minimize the experimental validation process. Subsequent site-directed mutagenesis of the residues involved in the major epitope, S22, led to the identification of amino acids that are important for allergen-IgE interactions. Identification of immunodominant regions of Pen c 13 may facilitate the development of specific peptide-based diagnostics and possibly lead to therapies for mold allergy.

## Materials and Methods

### Patients' sera

Informed consent was obtained from all patients and volunteers. Our study was approved by the Ethics Committee of the National Taiwan University Hospital. Sera from 80 patients with mold allergy and control sera from 30 healthy volunteers were used in this study. Sera were collected at the National Taiwan University Hospital (Taipei, Taiwan) and stored in aliquots at −80°C until use. The allergic response was confirmed by clinical history and diagnosis, and characterized by measuring specific IgE reactivity using the ImmunoCAP® system (Thermo Fisher Scientific, Uppsala, Sweden). Mold-allergic patients were initially included on the basis of an ImmunoCAP score for the crude extract of *Penicillium notatum* recognized by human IgE antibodies≥class 2 (0.70 kU/l). Among these patients a total of 10 individuals who had a positive skin prick test (Method S1) response to Pen c 13 were selected to detect IgE-binding epitopes within Pen c 13.

### Purification of His-tagged rPen c 13

rPen c 13 was purified as described previously [6]. Briefly, the cDNA encoding mature Pen c 13 was obtained from a *P. citrinum* DNA library by polymerase chain reaction (PCR), and the amplified fragment was ligated into the vector pQE 30 (Qiagen, Chatsworth, CA, USA) to generate an expression plasmid for N-terminally His_6_-tagged Pen c 13 (pQE-30/Pen c 13), which was then used to transform *E. coli* M15 cells (Qiagen). Cultures of transformed cells were grown to an OD_600_ value of 0.6, induced with 1 mM isopropyl-β-D-thio-galactoside (IPTG; Sigma-Aldrich), and harvested after 2 h. The His-tagged rPen c 13, isolated as inclusion bodies, was bound to a Ni^2+^-chelate affinity column, washed with binding buffer (20 mM Tris-HCl [pH 7.9], 0.5 M NaCl, 8 M urea, and 5 mM imidazole), and then eluted with elution buffer (20 mM Tris-HCl [pH 7.9], 0.5 M NaCl, 8 M urea, and 60 mM imidazole). Finally urea was removed by sequential dialysis with reducing urea concentrations from 8 M to 4 M, 2 M, 1 M, and 0 M against phosphate-buffered saline (PBS). The dialyzed rPen c 13 was concentrated using the concentrators with 10 kDa cut-off membrane (Amicon Ultra, Millipore). Protein concentrations were determined using a bicinchonic acid protein assay reagent kit (Pierce, Rockford, IL, USA) using BSA as the standard. The purity of rPen c 13 was verified by Coomassie Brilliant Blue staining after SDS-PAGE and mass spectrometric analysis.

### Specific IgE quantification

The detection of specific IgE antibodies reactive with Pen c 13 was examined by enzyme-linked immunosorbent assay (ELISA) using sera from 80 patients and 30 healthy controls. Microtiter plates (Costar Inc., Cambridge, USA) were coated with rPen c 13 by incubating with a 0.5-µg/ml solution of rPen c 13 in PBS. Each serum sample was serially diluted in PBS/0.1% Tween-20 and added to plates, which were then incubated for overnight at 4°C. Bound IgE was detected by incubating sequentially with alkaline phosphatase-labeled anti-human IgE antibodies (Biosource International, Camarillo, CA, USA) and a *p*-nitrophenyl phosphate substrate. Plates were allowed to stand in the dark for 30 min before reading at 405 nm. The cutoff was determined as two times the mean of the 30 controls. To adjust for inter-plate variability, normalized ELISA values were calculated by dividing the optical density (OD) of the test samples by the mean OD of the reference sera on each plate.

### Peptide purification and identification

Pen c 13 peptides were obtained through enzymatic and chemical cleavage. rPen c 13 was digested overnight at 37°C with lysyl endopeptidase (EC 3.4.21.50), trypsin (EC 3.4.21.4), chymotrypsin (EC 3.4.21.1), or Glu-C endopeptidase (EC 3.4.21.19) at an enzyme/substrate ratio of 1∶50. Lysyl endopeptidase, trypsin, and chymotrypsin digestions were carried out in 0.1 M pyridine/acetate/collidine (pH 8.2), and Glu-C endopeptidase digestions were performed in 0.1 mM calcium chloride and 0.1 M pyridine/acetate (pH 6.5). The resulting peptides were fractionated by reversed-phase high-performance liquid chromatography (HPLC) on a Beckman ODS (C18) column (300 Å, 4.6×250 mm) using a linear gradient of 0–44% acetonitrile in 0.06% trifluoroacetic acid and a flow rate of 1 ml/min. Cyanogen bromide (CNBr) cleavage was performed at 37°C for 24 h in 70% formic acid with a 100-fold molar excess of CNBr over methionine residues. After cleavage, the fragments were separated by HPLC on a YMC-Pack C4 column (300 Å, 4.6×150 mm) using the same elution system as for the Beckman column. Peptide elution was monitored at 220 nm, and all fractions were collected and analyzed for peptide mass and purity using a Procise ABI 494 protein sequencer (Applied Biosystems, Foster City, CA, USA) and a QSTAR XL mass spectrometer (Applied Biosystems).

### Dot-blot immunoassays

Approximately 30 pmol each of lysyl endopeptidase-, trypsin-, chymotrypsin-, Glu-C endopeptidase-, and CNBr-cleaved peptides from rPen c 13 was spotted onto a polyvinylidene fluoride (PVDF) membrane. A total of 24 peptides covering most of the Pen c 13 sequence were screened for their ability to bind human IgE in individual serum samples from allergic subjects. Bound antibodies were detected using alkaline phosphatase-conjugated goat anti-human IgE (Biosource) and the CDP-Star detection system (GE Healthcare), according to the manufacturer's instructions.

### IgE-binding inhibition assay

To evaluate the major epitopes S16 and S22, using three individual patient sera (patient 1, 7, and 10), with a high, medium and low ranges of the ELISA OD values were previously incubated overnight at 4°C with different concentrations of S16, S22, or both. For inhibition control, a pool of sera from the 10 patients was previously incubated overnight at 4°C with different concentrations of rPen c 13 as a positive control or albumin as the negative one. Microtiter plates (Costar Inc., Cambridge, USA) were coated with rPen c 13 by incubating for overnight at 4°C with 100 µl of a 5-µg/ml solution of rPen c 13 in PBS, then incubated for overnight at 4°C with aliquots of separate sera (diluted 1∶10) that had previously been incubated with different concentrations of peptides or proteins as mentioned above. The amount of specific IgE bound to the wells was determined as described above.

### Computational matching of epitopes

To assist in the identification of potential residues that are critical in IgE binding, we utilized an epitope conservancy analysis tool. This tool was implemented as a component of the Immune Epitope Database and Analysis Resources (IEDB) [39] and was used for predicting B-cell epitopes. Amino acid property scales for predicting antigenic determinants were developed based on Bepipred Linear Epitope Prediction (BepiPred), which predicts the location of linear B-cell epitopes using a combination of a hidden Markov model and a propensity scale method [35].

### Molecular modeling of Pen c 13

Pen c 13 was modeled based on the structure of the alkaline serine protease from *Lecanicillium psalliotae*, Ver112 (Protein Data Bank code 3F7M), which is the mostly closely related to Pen c 13 (highest BLAST score value) in the structure database [44]. To further examine the Pen c 13 structure, we generated a three-dimensional (3D) model structure using the SWISS-MODEL structure homology-modeling server [45]. The images were obtained and rendered with PyMOL software.

### Expression and purification of the GST fusion rPen c 13 peptides, (A^243^–K^274^), (A^243^–A^260^) and (T^261^–K^274^)

The region of the Pen c 13 targeted for (A^243^–K^274^), (A^243^–A^260^) and (T^261^–K^274^), is amplified using pQE-30/Pen c 13 as a template and the PCR with oligonucleotide primers (F243 *Bam*HI, 5′-GGATCCGCTCTCGAGGGCGTGTCAGCT-3′; R274 *Eco*RI, 5′-GAATTCTTACTTGCTTGTAGTGCCAGA-3′; R260 *Eco*RI, 5′-GAATTCTTAAGCGAGCTGGACAATACG-3′; F261 *Bam*HI, 5′-GGATCCACCTCGTCAATCTCTAGGGCT-3′) designed specifically for amplification of the piece of cDNA of interest. Incorporating restriction sites into the primers facilitates subcloning into the multiple cloning site of the pGEX-2T vector. The PCR products were gel-purified and then ligated into the pGEX-2T expression vector (GE Healthcare) at *Bam*HI and *Eco*RI sites. The fidelity of the cloned sequence was confirmed by DNA sequencing. For production of recombinant proteins, all plasmids were transformed into *Escherichia coli* strain *BL-21* (DE3) (Stratagene, La Jolla, CA). Cultures of transformed cells were grown to an OD_600_ value of 0.6, induced with 1 mM IPTG, and harvested after 4 h. Cells were resuspended in binding buffer (PBS, pH 7.4, containing 1% Triton X-100) and sonicated on ice for 5 min, after which the suspension was centrifuged and the supernatant was loaded onto a Glutathione Sepharose 4 Fast Flow column (GE Healthcare) pre-equilibrated in binding buffer. After adsorption, the non-bound material was removed by washing with binding buffer, and bound protein was eluted using elution buffer (50 mM Tris/HCl, pH 8.0, containing 10 mM reduced glutathione). Eluted fractions were collected and analyzed by SDS-PAGE.

### Mutant clones

The gene fragment for wild-type (WT) Pen c 13 (T^261^–K^274^) was cloned into the pGEX-2T vector as described above and used as a template for the preparation of all Pen c 13 (T^261^–K^274^) mutants. These mutants were generated using the QuikChange Site-Directed Mutagenesis kit (Stratagene, La Jolla, CA). All mutated genes were confirmed by direct DNA sequencing. The primers used for mutagenesis were as follows:

S269A: 5′-ATCTCTAGGGCTCCCGCTGGCACTACAAGCAAG-3′

G270A: 5′-TCTAGGGCTCCCTCTGCCACTACAAGCAAGTAA-3′

T271A: 5′-AGGGCTCCCTCTGGCGCTACAAGCAAGTAAGAA-3′

T272A: 5′-GCTCCCTCTGGCACTGCAAGCAAGTAAGAATTC-3′

S273A: 5′-CCCTCTGGCACTACAGCCAAGTAAGAATTCATC-3′

K274A: 5′-TCTGGCACTACAAGCGCGTAAGAATTCATCGTG-3′

The nucleotide sequences were obtained from GenBank (accession no. AF084546); the mutated sites are underlined. The nucleotide sequences of all clones were confirmed by DNA sequencing. All GST fusion rPen c 13 (T^261^–K^274^) mutants were expressed and purified as described above.

### SDS-PAGE and immunoblotting with IgE

GST fusions of WT rPen c 13 (A^243^–K^274^), (A^243^–A^260^) and (T^261^–K^274^), and mutants of rPen c 13 (T^261^–K^274^) were separated by SDS-PAGE along with GST only (control). For specific IgE immunodetection, resolved GST fusion peptides were transferred to a PVDF membrane, which was then blocked with skim milk, and incubated with serum (diluted 1∶10) from mold-allergic patients. Bound human IgE was detected using horseradish peroxidase-conjugated goat anti-human IgE antibodies (BioSource). Bands were visualized using enhanced chemiluminescence reagents (Millipore) and autoradiography. Densitometric analysis was performed using the LabWorks software (UVP BioImaging Systems, Upland, CA, USA). The relative density of the bands normalized to loading control.

## Results

### Determination of Pen c 13 IgE reactivity in patient sera by ELISA

**Figure 1:**
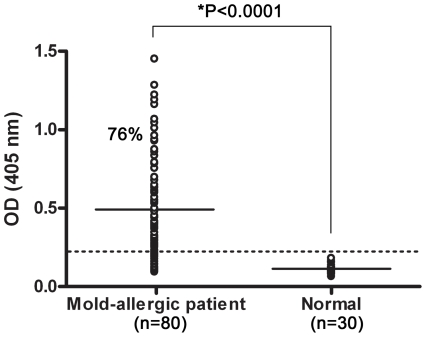
IgE-reactivity of human sera against rPen c 13. The binding of rPen c 13 to serum IgE from 80 patients allergic to molds and 30 nonatopic subjects was tested by ELISA. Each data point represents an individual patient or healthy control. The horizontal dotted line shows the cutoff of the reaction, defined as two times the mean value of the healthy control group. Percentage values represent the overall frequency of positive responders among mold-allergic patients. The mean values are indicated by horizontal bars. Significance for unpaired comparisons of mean values between two groups was calculated using a two-tailed Mann-Whitney *U* test. The significance level was specified as α=0.05. *P<0.0001.

We have previously reported that Pen c 13 is a major allergen in mold-allergic patients [6]. To obtain sera contained higher specific IgE titers, we first analyzed 80 patients with mold allergy for Pen c 13-binding IgE by ELISA. The sera from 10 patients displaying high IgE reactivity were used to identify potential B-cell epitopes in Pen c 13.The distribution of Pen c 13-specific IgE values in all patients is shown in Figure 1. Seventy-six percent (61/80) of mold-allergic patients showed IgE binding to rPen c 13 at levels exceeding the two-fold mean of controls. Some characteristics of 10 patients selected for further evaluation and associated serum total IgE levels are shown in Table 1.

**Table 1:** Characterization of sera from patients with positive IgE binding to rPen c 13.

Patient No.	Sex	Age(y)	Symptoms	Serum total IgE (kU/L)	Pen c 13 ELISA (OD)	ImmunoCAP score
1	M	66	AS	951	1.45	+3
2	M	56	AS,AR	1565	1.28	+2
3	M	41	AS,UR	411	1.22	+2
4	M	24	AR	120	1.19	+2
5	M	22	AD	>2000	1.16	+4
6	M	28	AS,AR,AD	782	1.07	+2
7	F	22	AR,AD	>2000	1.04	+2
8	F	10	AR,AD	>2000	1.01	+3
9	M	33	AS,AR,AD	>2000	0.96	+3
10	M	20	AS,AR,AD	>2000	0.94	+3

AD, atopic dermatitis; AR, atopic rhinitis; AS, asthma; UR, urticaria.

### Identification of IgE-binding epitopes of rPen c 13

**Figure 2:**
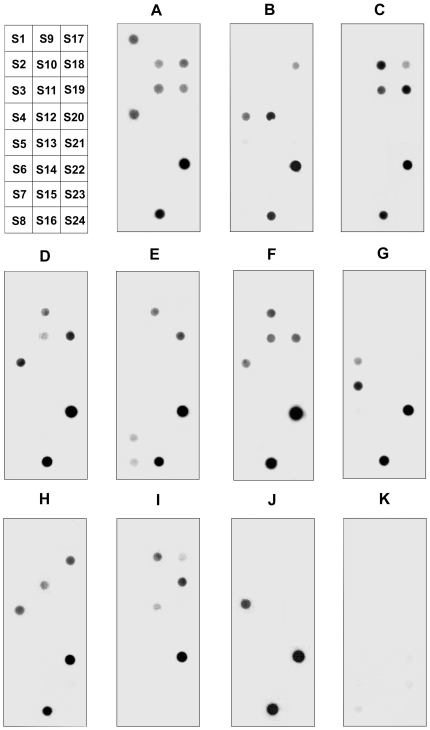
Mapping of IgE-binding epitopes by dot-blotting. Twenty-four overlapping peptides spanning almost the entire sequence of Pen c 13 were tested for reactivity toward serum IgE from 10 mold-allergic patients (A–J) and nonatopic individual (K). The diagram on the top-left shows the pattern of peptides on the blots.

Peptide fractions corresponding to 24 peptides covering almost the entire amino acid sequence of Pen c 13 were produced by cleavage for use in dot-blot screens of sera from 10 Pen c 13-allergic individuals (A–J) (Fig. 2). The intensity of IgE binding and epitope recognition by serum IgE varied substantially among individuals. Twelve different IgE-binding determinants were found among the 24 peptides. Antibody-binding regions were identified in peptides S1, S4, S5, S7, S8, S10, S11, S12, S16, S18, S19 and S22, corresponding to residues 1–19, 30–51, 40–52, 58–85, 86–90, 95–117, 99–120, 122–140, 148–166, 181–204, 197–224 and 243–274, respectively. Table 2 summarizes the IgE reactivity of these 12 IgE-binding epitopes and their respective positions in the Pen c 13 molecule. Epitopes S16 and S22 were recognized by serum IgE from 90% (9/10) and 100% (10/10) of the mold-allergic patients, and both epitopes were classified as dominant epitopes in the sampled population. Other epitopes were recognized by serum IgE in 10–70% of the 10 patients tested. The control serum sample (K) showed no IgE reactivity (Fig. 2).

**Table 2:** Characteristics of peptide fragments used for human IgE-binding dot-blot assays.

Peptide No.	IgE reactivity (%)	N-terminal sequence	Mass (Da) [M+H]^+^	Position in Pen c 13
**S1**	10	ANVVQ	2013.0	Ala^1^-Lys^19^
**S2**	–	TGTTS	1008.4	Thr^21^-Asp^29^
**S3**	–	TYDST	1336.5	Thr^27^-Tyr^39^
**S4**	70	STAGE	2154.0	Ser^30^-Asp^51^
**S5**	10	GVDTG	1362.6	Gly^40^-Phe^52^
**S6**	–	FGGRA	1406.4	Phe^52^-Asp^64^
**S7**	10	WGTNV	2814.0	Trp^58^-Lys^85^
**S8**	10	YGVAK	537.4	Tyr^86^-Lys^90^
**S9**	–	KATLV	829.0	Lys^91^-Lys^98^
**S10**	60	VAVKV	2220	Val^95^-Trp^117^
**S11**	50	VLGAD	2121	Val^99^-Lys^120^
**S12**	20	AKSRG	1970	Ala^122^-Glu^140^
**S13**	–	YVMNM	1462.0	Tyr^131^-Lys^143^
**S14**	–	SKAVN	1691.0	Ser^142^-Phe^158^
**S15**	–	AVNDA	1071.0	Ala^144^-Lys^154^
**S16**	90	AAANV	1818.0	Ala^148^-Glu^166^
**S17**	–	NASNS	1105.1	Asn^169^-Glu^180^
**S18**	50	VCTIA	2442.0	Val^181^-Asp^204^
**S19**	60	TNFGS	2771.0	Thr^197^-Leu^224^
**S20**	–	SGTSM	1489.0	Ser^225^-Tyr^240^
**S21**	–	AAPHV	1222.2	Ala^230^-Met^242^
**S22**	100	ALEGV	3438.0	Ala^243^-Lys^274^
**S23**	–	IVQLA	1174.0	Ile^256^-Arg^266^
**S24**	–	LLYNG	905.5	Leu^275^-Val^282^

The purity of the peptides was assessed by N-terminal sequencing.

N-terminal residues were determined by Edman degradation.

Mass was determined using a QSTAR XL mass spectrometer.

### Confirmation of the major IgE-binding epitopes by inhibition ELISA

**Figure 3:**
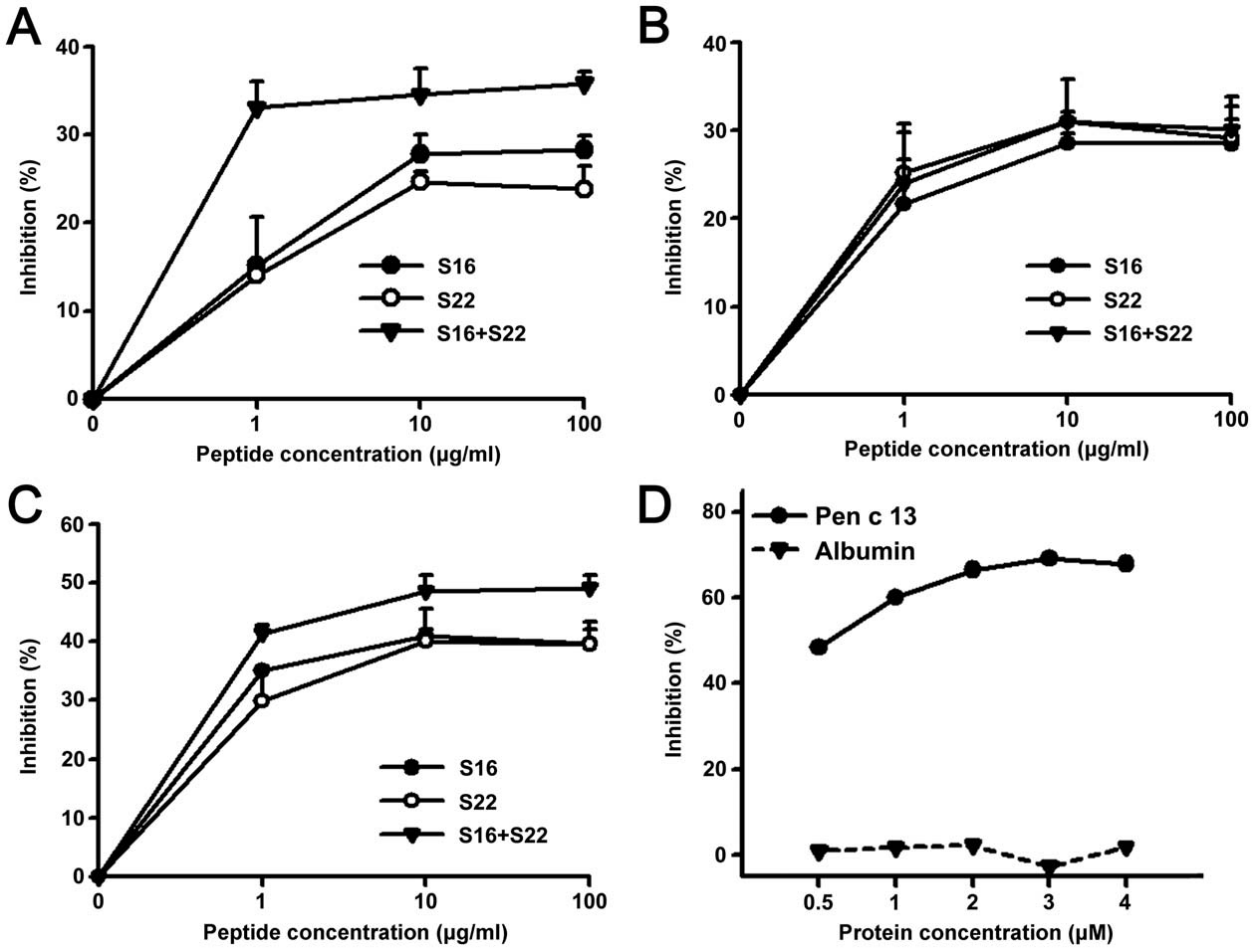
ELISA inhibition of S16 and S22 with sera from Pen c 13-allergic patients. A–C, Sera from three Pen c 13-reactive patients (1∶10 dilution) were pre-incubated with different concentrations of purified S16 (•), S22 (○), or S16 and S22 (▾) overnight at 4°C. The pre-incubated serum samples were transferred to rPen c 13-coated wells, and bound IgE was analyzed by ELISA. The percentage of binding inhibition after pre-incubation with the different inhibitors was calculated as [(OD−OD inhibitor)/OD]×100, where OD (optical density) and OD inhibitor indicate the absorbance values obtained without and with inhibitors before incubation, respectively. Samples were analyzed in triplicate. Error bars indicate the standard deviations of the measurements. D, Various concentrations of either rPen c 13 or albumin were pre-incubated with a pool of serum from 10 Pen c 13-reactive patients.

To analyze the relevance of IgE-binding sequences identified by dot-blotting, we performed IgE ELISA inhibition experiments. Three serum samples that recognized the epitopes S16 and S22 of Pen c 13 in dot-blots were used for ELISA inhibition analysis. Sera from patients preincubated with different concentrations of the peptide (S16, S22 or S16+S22) were evaluated for IgE binding to rPen c 13. The results showed that IgE reactivity with Pen c 13 was inhibited in a concentration-dependent manner by epitope S16 and/or S22 (Fig. 3A–C). For inhibition control, using Pen c 13 as a positive control and albumin as a negative one with a pool of sera were carried out to compare the inhibition capacity. As shown in Fig. 3D, maximal inhibition reaching 69.15% was found at 3 µM concentration of rPen c 13. Taken together, these results demonstrate that S16 and S22 are the major epitopes of Pen c 13.

### Molecular modeling of the location of IgE-binding epitopes in Pen c 13

**Figure 4:**
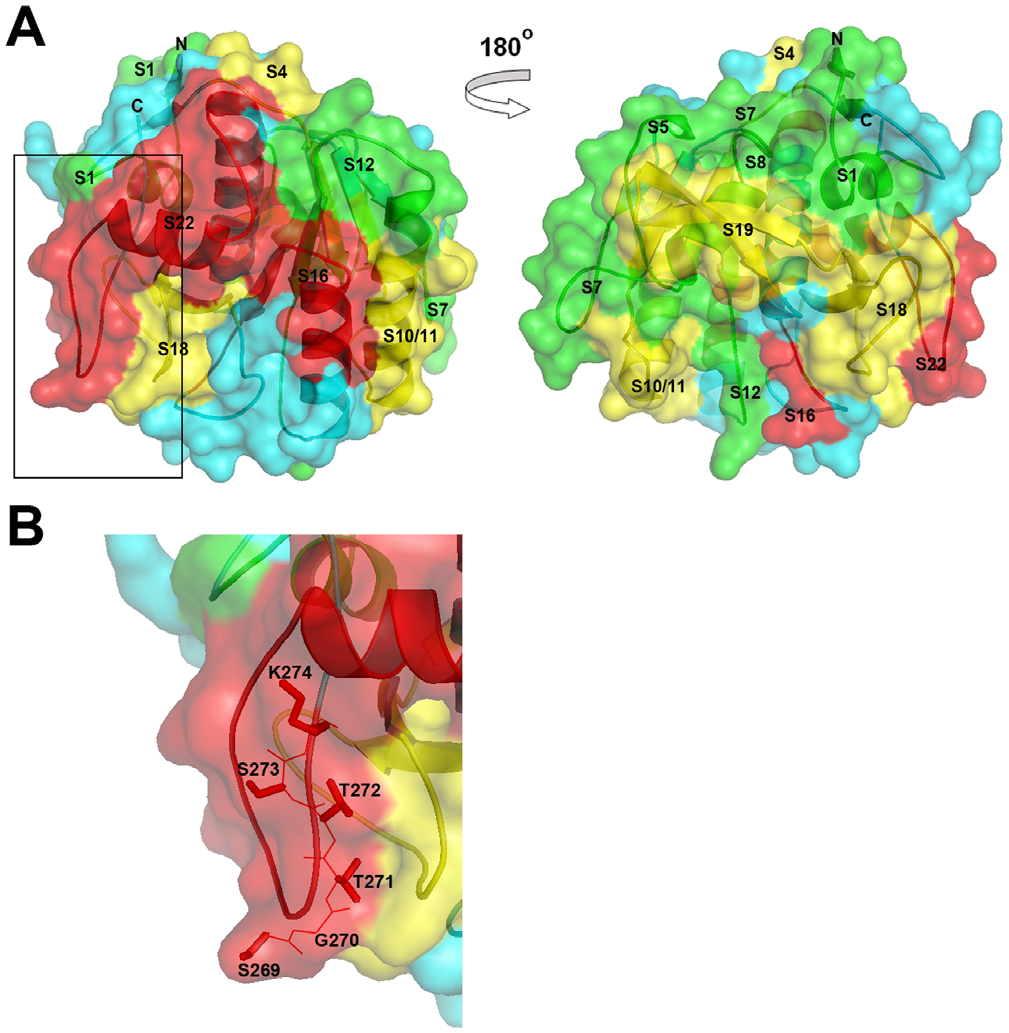
Molecular models and the immunodominant IgE-binding epitope of Pen c 13. A, Surface and ribbon diagrams of the Pen c 13 model. The different strengths of IgE-binding epitopes are colored green (weak), yellow (intermediate), and red (strong). Different levels of IgE-binding intensity are defined as: weak (10–20%), intermediate (50–70%), and strong (90–100%). B, Stick diagram of Pen c 13 residues 269–274.

According to the results of IEDB prediction, shown in Table 3, the most critical residues involved in IgE binding are located in the C-terminus of the S22 peptide, especially the final six-residue region, which had higher prediction scores. To obtain more detailed information on the binding of IgE to Pen c 13, we built a 3D structure of Pen c 13 using the crystal structure of the cuticle-degrading protease, Ver112, secreted by *Lecanicillium psalliotae*, as a suitable template (PDB ID: 3F7M). The identity and similarity between the target and template were 48% and 67%, respectively. As shown in Figure 4A, the model consists of an α/β core flanked by several amphipathic helices and anti-parallel β sheets. IgE reactivities is depicted by colors based on their intensity as follows: negative (blue), weak (green), intermediate (yellow), and strong (red). Epitope S1 is situated on the N-terminal β-sheet, one turn, and an α-helix, whereas S18 and S19 are located on one turn and four β-sheets close to the C-terminus. Epitope S4 consists of two turns and one β-sheet adjacent to the N-terminal region, whereas S5, situated similarly, lacks one turn and part of the β-sheet, accounting for its marked reduction in IgE reactivity. Epitopes S7 and S8 consist of two turns and one α-helix. Epitopes S10, S11, and S12 consist of three turns, two β-sheets, and one α-helix; epitope S10/S11 is considered to contain the same allergenic sites (one turn and one helix), but S10 contains an extra β-sheet in its N terminus. Of the two immunodominant IgE epitopes, S16 forms α-helices and a β-sheet in the middle part of the protein, and S22 forms α-helices and turns in the extreme C-terminal domain. The current model of Pen c 13 reveals that the six residues predicted from IEDB are situated on a turn and are located on the protein surface (Fig. 4B). Thus, we suggest that the final six-residue region is an important antigenic region of Pen c 13.

**Table 3:** BepiPred Linear Epitope Prediction scores of each residue in the S22 peptide (residues 243–274 of Pen c 13).

Position	Residue	Score	Position	Residue	Score
**1**	A^243^	−0.092	**17**	L^259^	−0.487
**2**	L^244^	0.21	**18**	A^260^	−0.582
**3**	E^245^	0.25	**19**	T^261^	−0.222
**4**	G^246^	0.444	**20**	S^262^	0.155
**5**	V^247^	0.531	**21**	S^263^	0.309
**6**	S^248^	0.568	**22**	I^264^	0.757
**7**	A^249^	0.734	**23**	S^265^	1.057
**8**	G^250^	0.511	**24**	R^266^	1.263
**9**	N^251^	0.331	**25**	A^267^	1.336
**10**	A^252^	0.106	**26**	P^268^	1.397
**11**	C^253^	−0.321	**27**	S^269^	1.758
**12**	A^254^	−0.388	**28**	G^270^	1.789
**13**	R^255^	−0.85	**29**	T^271^	1.855
**14**	I^256^	−0.979	**30**	T^272^	1.938
**15**	V^257^	−0.896	**31**	S^273^	1.981
**16**	Q^258^	−0.728	**32**	K^274^	1.824

### Modulation of IgE reactivity by site-directed mutagenesis

**Figure 5:**
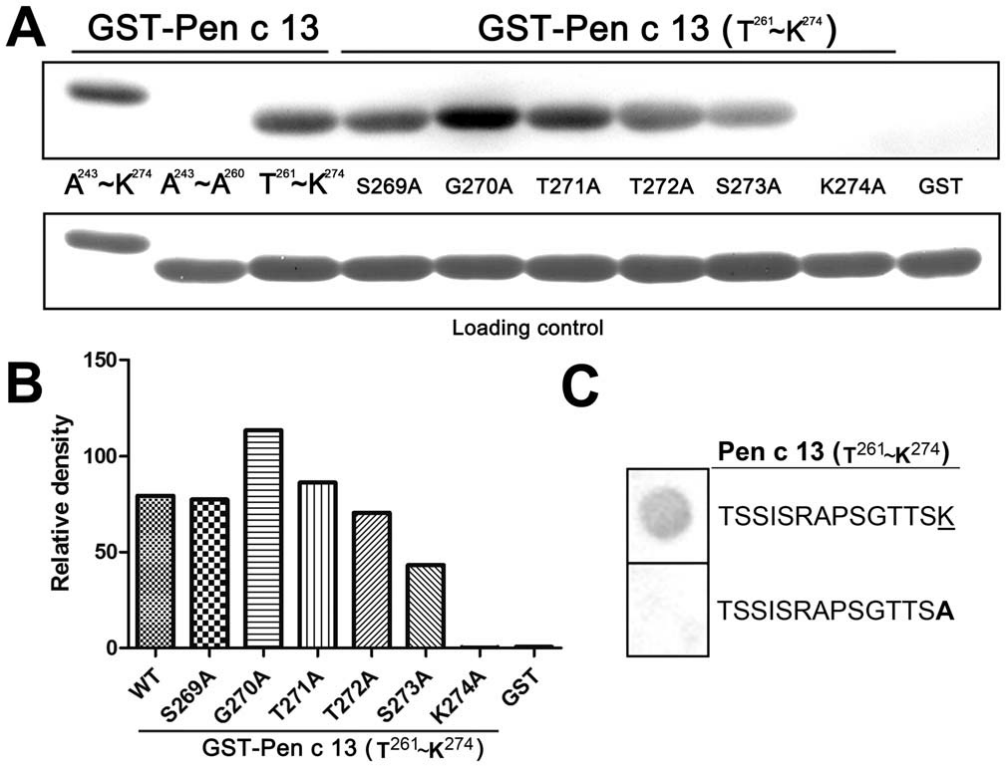
Analysis of IgE reactivity to GST fusion peptides and synthetic peptides. A, The data shown represent the results for patient 10. Immunoblots of purified GST fusion peptides Pen c 13 (A^243^–K^274^), (A^243^–A^260^) and (T^261^–K^274^); GST fusion Pen c 13 (T^261^–K^274^) mutant peptides S269A, G270A, T271A, T272A, S273A or K274A; and GST only, probed with serum from a Pen c 13-allergic patient. The protein blots were stained with Fast Green as a loading control. B, The histogram shows the quantitative densitometry of the bands. C, The critical amino acid for IgE-binding in Pen c 13 (T^261^–K^274^) was validated further using two synthetic peptides and tested for IgE-binding reactivity with pool serum from 10 allergic patients. The sequences of two synthetic peptides are listed, and the residue that was changed to alanine is *underlined*. Alanine substitution is shown in *boldface letter*.

To validate the IgE-binding region of Pen c 13 (A^243^–K^274^) and narrow down the candidate epitope residues predicted by the *in silico*-based method, we expressed and isolated the GST fusion peptides, rPen c 13 (A^243^–A^260^) and rPen c 13 (T^261^–K^274^). Immunoblotting unambiguously showed that IgE reactivity is located in the rPen c 13 (T^261^–K^274^) (Fig. 5A), consistent with *in silico* predictions. Using site-directed mutagenesis assays, we further confirmed the theoretical prediction of potential IgE-binding regions, which presumably exhibit both a protuberant local surface and an electrostatically active local molecular domain. Consistent with this presumption, the side chains of these predicted residues are all located on the polar half of the surface, as shown in Figure 4B. We therefore created the alanine (A) substitution mutants, S269A, G270A, T271A, T272A, S273A and K274A, corresponding to these six residues within the rPen c 13 (T^261^–K^274^) GST fusion, and assayed their ability to bind IgE. The result of the immunoblot strip in patient 10 was shown in Fig. 5A. The bands were quantified by densitometric analysis and shown as histogram (Fig. 5B). Of the six individual substitutions, the K274A mutation fully abolished IgE recognition; in contrast, the G270A mutation substantially increased IgE-binding capacity. In addition, pooled serum IgE from 10 patients was analyzed in the same manner, and the critical amino acids for IgE-binding were found to be the same as in patient 10 (data not shown). These results suggest that G270 and K274 are important for the IgE-binding activity of the Pen c 13 allergen. In order to validate the reduced IgE reactivity, we performed dot-blot immunoassay using two synthetic peptides (TSSISRAPSGTTSK and TSSISRAPSGTTSA), followed by probing with pool serum from 10 patients. The result is shown in Fig. 5C; no binding of the serum IgE was observed when the alanine was substituted at the lysine position.

## Discussion

Identification of IgE-binding epitopes might provide a better understanding of the functional role that allergens play in the disease and could have implications for immunodiagnosis and immunotherapy. A number of approaches are currently used to predict linear epitopes, including sequence-based prediction software and 3D structure-based prediction [46]. Such approaches, most of which are based on the physicochemical properties of the amino acids in the molecule, may provide a better understanding of antigen-antibody binding. Linear epitopes of antigens share some common properties: they are exposed to the surface of the allergenic molecule, making them easily accessible; they are localized to flexible regions; and they are composed of polar residues.

In the present study, we mapped IgE-binding epitopes using enzymatically/chemically cleaved fragments to obtain more detailed information on the binding of IgE to the Pen c 13 molecule. Cleaved peptides covering most of the sequence of Pen c 13 were analyzed by dot-blotting using sera from mold-allergic patients. Twelve distinct IgE-binding epitopes distributed throughout the entire molecule were found, although additional epitopes may also exist. Only a few epitopes may have been missed because epitopes are located on cleavage sites and small gaps in the sequence coverage. In molecular modeling studies, these regions were predicted to lie on the protein surface and contained mostly solvent-exposed residues. The results suggest that IgE predominantly recognizes linear epitopes, the S22 peptide (residues 243–274), accounts for a high percentage of Pen c 13-specific IgE binding. In a 3D model of Pen c 13 based on the structure of *Lecanicillium psalliotae* Ver112, the IgE-binding determinant ^261^TSSISRAPSGTTSK^274^ assumes a loop-like structure on the surface of the protein. This finding is consistent with the known importance of loop-like structures in IgE binding and the reported allergenicity of other allergens, such as transaldolase, a fungal allergen whose IgE-reacting fragment is located in a loop-like structure of the C-terminal region [47]; the dust mite allergen Der f 7, whose IgE-binding epitope forms a loop-like structure on the surface of the protein [48]; the olive pollen allergen Ole e 9, whose B-cell epitopes within the C-terminal domain are mainly located in loops [49]; the mugwort pollen allergen Art v 1, whose non-linear IgE epitopes are found within exposed loop regions harboring lysine residues [50]; and the bovine milk allergen β-lactoglobulin, whose strongest IgE epitope is located in a loop [51]. Furthermore, IgE interactions with hyaluronidase, a major bee venom allergen, may also involve a surface-exposed loop-like IgE determinant [52].

Using computational prediction, we further narrowed the responsible sequence to a six-residues region (^269^SGTTSK^274^) and generated six mutants with single alanine substitutions to test the role of the predicted residues. The K274A mutation largely abolished IgE-binding ability, reflecting charge and/or size differences between lysine and alanine, implying that K274 is a major contributor to this interaction. Dramatic effects of single amino acid substitutions on the IgE-binding properties of allergens have been previously reported [34], [48], [53]–[56]. Findings from these studies suggest the importance of surface charged residues in the IgE binding and allergenicity of an allergen. For instance, it was found that the IgE-binding epitopes of the dust mite allergen Der f 13 [54], the latex allergen Hev b 6.02 [53], and the cockroach allergen Bla g 2 [56] are confined to lysine residues exposed on the surface of the allergens, a result similar to our observations for Pen c 13.

IgE antibodies in sera of allergic subjects are considered to be polyclonal and may recognize several different epitopes on an allergen. We modeled the K274A and G 270A mutant structures and then compared to the native structure, which shows no difference in the entire molecule. In fact, a previous study indicated that mutation of single epitope within whole allergen had little influence on IgE binding, but a combination of several mutated epitopes in the entire molecule was able to reduce IgE reactivity dramatically [57]. Moreover, in an animal model intranasal application of genetically produced hypoallergenic fragments of Bet v 1 produced mucosal tolerance that was seen with the complete Bet v 1 allergen [58]. Clinical studies using short peptides may have an advantage of being unable to activate mast cell and basophil by cross-link allergen-specific IgE while maintaining their ability to modify T-cell and/or B-cell responses resulting in reduction in Th2 cytokines and late phase reaction on challenge [59]–[62]. Based on the above studies, we conclude that the K274A mutant of Pen c 13 with abolished IgE-binding activity may be a candidate for immunotherapy of mold allergies.

Genetically modified recombinant allergen derivatives designed to reduce allergenic activity induce blocking antibodies that inhibit the binding of allergic patients' IgE antibodies to allergens and hence represent a B cell-based approach to the treatment of allergies [63], [64]. Moreover, these molecules may also preserve the repertoire of allergen-specific T cell epitopes, and thus can be utilized for targeting T cells. The use of peptide fragments corresponding to T-cell epitopes to induce immunologic tolerance has been reported in experimental models of allergic disease [61], [65]–[68]. Our approach is based on a clear rationale and measurable parameters for the development of recombinant hypoallergen derivatives. However, T cell epitope within S22 peptide still remains to be elucidated by *in vitro* and *in vivo* assay.

In conclusion, we characterized a dominant linear IgE epitope of Pen c 13 in this study, identifying residues 261–274 as an immunodominant epitope. Gly270 and Lys274 are among the critical core amino acids of Pen c 13 recognized by human IgE antibodies. These residues are located on a loop-like structure at or near the surface of Pen c 13. The results obtained from the present study provide information on the molecular and structural features that define the allergenicity of the Pen c 13 allergen and can be used for developing better diagnostic tools and effective immunotherapeutic strategies for the clinical management of fungal allergy.

## Supporting Information

Method S1 **Skin prick test (SPT).** An SPT was performed on 10 patients who had serum IgE reactive with Pen c 13 in a specific IgE ELISA and a history of mold allergy. The skin testing was performed using 50 µl of purified Pen c 13 from *Penicillium citrinum* at concentrations of 10 mg/ml and 100 mg/ml in physiological saline (0.9% NaCl) or physiological saline solution alone as a negative control. Skin reactions (wheals and erythema) were recorded 15 min after prick. Wheals with a diameter of at least 1.5 mm greater than that produced by the negative control were regarded as positive responses.
(DOC)
