# Supplementary material for: Identification of Critical Amino Acids in an Immunodominant IgE Epitope of Pen c 13, a Major Allergen from Penicillium citrinum
Source: PLoS One. 2012 Apr 10;7(4):e34627. doi: 10.1371/journal.pone.0034627 (PMC3323554; doi:10.1371/journal.pone.0034627)
Supplement: Method S1 — Skin prick test (SPT). An SPT was performed on 10 patients who had serum IgE reactive with Pen c 13 in a specific IgE ELISA and a history of mold allergy. The skin testing was performed using 50 µl of purified Pen c 13 from Penicillium citrinum at concentrations of 10 mg/ml and 100 mg/ml in physiological saline (0.9% NaCl) or physiological saline solution alone as a negative control. Skin reactions (wheals and erythema) were recorded 15 min after prick. Wheals with a diameter of at least 1.5 mm greater than that produced by the negative control were regarded as positive responses. (DOC) [file pone.0034627.s001.doc]

**Supplemental Methods**

***Skin prick test (SPT)***

An SPT was performed on 10 patients who had serum IgE reactive with Pen c 13 in a specific IgE ELISA and a history of mold allergy. The skin testing was performed using 50 µl of purified Pen c 13 from *Penicillium citrinum* at concentrations of 10 mg/ml and 100 mg/ml in physiological saline (0.9% NaCl) or physiological saline solution alone as a negative control. Skin reactions (wheals and erythema) were recorded 15 min after prick. Wheals with a diameter of at least 1.5 mm greater than that produced by the negative control were regarded as positive responses.
